# Bioconversion of cheese whey permeate into fungal oil by *Mucor circinelloides*

**DOI:** 10.1186/s13036-018-0116-5

**Published:** 2018-11-14

**Authors:** Lauryn G. Chan, Joshua L. Cohen, Gulustan Ozturk, Marie Hennebelle, Ameer Y. Taha, Juliana Maria L. N. de Moura Bell

**Affiliations:** 1 Department of Food Science and Technology, University of California, Davis, One Shields Avenue, Davis, CA 95616 USA; 2 Department of Biological and Agricultural Engineering, Davis, One Shields Avenue, Davis, CA 95616 USA

**Keywords:** Oleaginous fungus, Fermentation, Microbial lipid, Biomass, Whey permeate, Bioconversion, Optimization

## Abstract

**Background:**

Oleaginous fungi are efficient tools to convert agricultural waste streams into valuable components. The filamentous fungus *Mucor circinelloides* was cultivated in whey permeate, a byproduct from cheese production, to produce an oil-rich fungal biomass. Response surface methodology was used to optimize the fermentation conditions such as pH and temperature for increased biomass yield and lipid accumulation. Quantification and characterization of the fungal biomass oil was conducted.

**Results:**

Upstream lactose hydrolysis of the whey permeate increased the biomass yield from 2.4 to 7.8 (g dry biomass/L) compared to that of non-hydrolyzed whey permeate. The combination of low pH (4.5) and pasteurization minimized microbial competition, thus favoring fungal growth. A central composite rotatable design was used to evaluate the effects of temperature (22.4–33.6 °C) and a lower pH range (3.6–4.7) on biomass yield and composition. The highest biomass yield and oil content was observed at high temperature (33.6 °C), while the pH range evaluated had a less pronounced effect. The predictive model was validated at the optimal conditions of 33.6 °C and pH 4.5. The fungal biomass yield plateaued at 9 g dry cell weight per liter, while the oil content and lipid yield reached a maximum of 24% dry biomass and 2.20 g/L, respectively, at 168 h. Triacylglycerides were the major lipid class (92%), which contained predominantly oleic (41%), palmitic (23%), linoleic (11%), and γ-linolenic acid (9%).

**Conclusions:**

This study provided an alternative way of valorization of cheese whey permeate by using it as a substrate for the production of value-added compounds by fungal fermentation. The fatty acid profile indicates the suitability of *M. circinelloides* oil as a potential feedstock for biofuel production and nutraceutical applications.

**Electronic supplementary material:**

The online version of this article (10.1186/s13036-018-0116-5) contains supplementary material, which is available to authorized users.

## Background

The increasing production and concomitant underutilization of industrial waste streams and food coproducts is a growing threat to the environmental and financial sustainability of the food industry. The dairy industry not only consumes a large volume of water, but also produces a high amount of effluent per unit of production [1]. Cheese whey, a major coproduct from cheese making, is primarily used to produce whey protein concentrates. However, the production of whey protein concentrates by ultrafiltration generates another stream, known as whey permeate (WP), which is composed of minerals (6–20%), proteins (0.5–3%), a high content of lactose (70–90%), and a small amounts of lipids (< 2%) on a dry weight basis. Current utilization practices of WP include land spreading, incorporation into animal feed, lactose crystallization or selling as dry permeate powder [2, 3]. As with many other agricultural streams, WP has a high biological oxygen demand of 40,000–48,000 mg/L and chemical oxygen demand of (COD) of 80,000–95,000 mg/L [2]. Therefore, WP cannot be disposed of in the environment without previous pretreatments to reduce its BOD and COD, representing costs for the processors. Consequently, alternative strategies for the cost-effective use of WP are needed for the dairy industry to decrease economic losses and environmental pollution.

Recently, oleaginous microorganisms, such as microalgae, yeast, and filamentous fungi, have been identified as efficient tools in converting agricultural waste streams into valuable food, feed and fuel ingredients [4–7]. These oleaginous microorganisms can accumulate over 20% of their biomass as lipids. Their fast growth potential and high lipid productivity make them a viable choice for the conversion of many nutrient-rich streams into added-value compounds [8]. Thus far, industrial adoption has typically been hindered by economically infeasible separation costs of the oleaginous cells from the media [9]. Hence, the use of filamentous fungi, which can grow into pellets or agglomerates that are easily dewatered by simple screening strategies, becomes attractive when compared with other oil-producing microorganisms such as algae and yeasts. The possibility of avoiding the use of high-energy separation techniques such as centrifugation can potentially reduce downstream processing costs, thus improving the economic feasibility of fungal biomass derived products. As an example, *Rhizopus microsporus* has been successfully cultivated on thin stillage, a by-product from the ethanol fermentation, from lab- to pilot-scale (1300 L) producing a biomass with high oil content [10]. In addition to being a source of oil, fungal biomasses can also be a source of crude protein containing several essential amino acids.

Nitrogen limitation resulting from a high carbon to nitrogen ratio can stimulate lipid accumulation when using filamentous fungi. When cells run out of a key nutrient, such as nitrogen, excess carbon substrate continues to be assimilated by the cells for lipid synthesis and oil droplets containing triacylglycerides (TAG) are formed [11]. The TAG fraction of most filamentous fungal oils is similar to that of conventional plant oils, indicating its potential use as a favorable oil feedstock for the food and fuel industry [12]. The biotechnology industry has also focused on the ability of oleaginous fungi to convert agricultural waste streams and residues into specialty lipids containing polyunsaturated fatty acids (FAs) of nutraceutical and pharmaceutical importance, such as γ-linolenic acid [13].

Optimized processing conditions enable tailoring the fungal biomass yield and composition for specific agricultural streams or food coproducts. Therefore, the present study was undertaken to optimize the use of a filamentous fungus (*Mucor circinelloides*) to convert the organic matter present in WP into an oil- and protein-rich biomass that can be further converted into value-added nutraceuticals, food, and fuel. The objectives of this study were to: 1) evaluate the effects of upstream lactose hydrolysis on sugar utilization and fungal growth; 2) determine the effects of heat treatments (pasteurization and sterilization) and cultivation pH on biomass yield and 3) simultaneously optimize processing conditions for increased biomass yield and lipid accumulation. A central composite rotatable design totaling 11 experimental conditions was used to identify the ideal combination of pH and temperature to improve biomass yield and composition. The fungal biomass was further characterized for protein, lipid content and composition to better identify potential industrial applications (e.g., food, feed, and fuel).

## Methods

### Production of bovine whey permeate and hydrolyzed whey permeate

Bovine whey was kindly provided by Hilmar Cheese Company, Inc. (Hilmar Inc., CA, USA). To assess the ability of the fungus to utilize different sugar sources (lactose, glucose, galactose), lactose was hydrolyzed prior to the fungal cultivation. A food grade fungal lactase (Bio-Cat Inc., Troy, Virginia, USA) derived from the fungus *Aspergillus oryzae* was used to hydrolyze lactose into β-D-galactose and α-D-glucose. The pH of 75–80 L of whey was adjusted to 4.5 with citric acid before adding 0.2% (*w*/*v*) of β-galactosidase and was stirred for 1 h at 50 °C. Lactose hydrolysis conditions were selected based on previous work [14]. Immediately following lactose hydrolysis, whey proteins were removed by ultrafiltration using a 10 kDa polyethersulfone membrane (Synder Filtration, Vacaville, CA, USA) to produce hydrolyzed whey permeate (HWP). After ultrafiltration, samples were pasteurized using a continuous UHT/HTST lab pasteurizer (MicroThermics, Raleigh, NC, USA) at 72 °C for 15 s and stored at − 20 °C until used. The HWP contained 6.3% solids, 0.26% protein, and 23.99, 21.63, and 0.11 g/L of glucose, galactose, and lactose, respectively.

### Total bacteria content of hydrolyzed whey permeate

pH and thermal treatments determine the growth and survival of most bacteria; therefore, microbiological analyses of the fermented pasteurized HWP at pH 4.5 and 6 were performed by the plate count agar method [15]. Samples were added to the agar plates (Difco, Detroit, MI, USA) and incubated at 34 °C for 1 day, after which the viable cell count was determined and expressed as colony-forming units per milliliter. All growth experiments were performed in triplicate. HWP was pasteurized as described above or sterilized in an autoclave at 121 °C for 20 min (Model 69,150, Tuttnauer, Hauppauge, NY, USA) to compare the efficiency of both heat treatments to inactivate microorganisms.

### Fungal strain and preculture preparation

The fungal strain *Mucor circinelloides f. lusitanicus (*ATCC® 1216B™) was obtained from the American Type Culture Collection (Manassas, VA, USA). Spore suspensions were prepared as described previously in potato dextrose broth and glycerol and stored at − 80 °C [16]. The spore suspension was used as the inoculum. A 1% *M. circinellodes* preculture (~ 1 mL inoculum per 100 mL of media) was prepared in a 250 mL baffled Erlenmeyer shake flask containing potato dextrose broth. The flask was placed on a rotary shaker (Excella E24 Incubator Shaker Series, New Brunswick Scientific, New York) at 34 °C with an agitation speed of 200 rpm for 24 h.

### Fungal cultivation and biomass recovery

Fungal cultivation was performed by aseptically transferring 5 mL of the preculture into 100 mL HWP in 250 mL baffled Erlenmeyer flasks. Samples were incubated in the rotary shaker according to the processing conditions described in the experimental design at constant stirring of 200 rpm. After fungal cultivation, the fungal biomass was harvested from the spent media using a simple mesh screen with 1 mm openings. Total dry weight of fungal biomass was determined by drying the wet biomass to constant weight in a vacuum oven (Jeio Tech, Model OV-11/12, Billerica, MA, USA) at 60 °C. Biomass was analyzed for oil and protein content, while the spent medium was analyzed for sugar and nitrogen content.

### Determination of biomass and lipid yields and coefficients

Yields and coefficients were determined according to Carota et al. [17] and Mitra et al. [16], with small modifications. The biomass yield was expressed as grams of dry biomass per liter of HWP (g/L). Lipid yield (∆P) was calculated according to Eq. (1) (g/L):

1 $$ \varDelta \mathrm{P}=\varDelta \mathrm{X}\ast \left({\mathrm{C}}_{\mathrm{L}}/100\right) $$

where ∆X is the biomass yield (g/L) and C_L_ is the intracellular lipid content (%).

Eqs. (2) and (3) were used to calculate the biomass and lipid yield coefficients (Y_X/S_ and Y_P/S_, respectively):

2 $$ {Y}_{X/S}=\varDelta \mathrm{X}/\varDelta \mathrm{S} $$

3 $$ {Y}_{P/S}=\varDelta \mathrm{P}/\varDelta \mathrm{S} $$

where ∆X and ∆P are the biomass and lipid yields (g/L), respectively, and ∆S is the amount of sugar consumed (g/L).

Eq. (4) was used to calculate the specific lipid yield (Y_P/X_):

4 $$ {\mathrm{Y}}_{\mathrm{P}/\mathrm{X}}=\kern0.5em \varDelta \mathrm{P}/\varDelta \mathrm{X} $$

where ∆P is the lipid yield (g/L) and ∆X is the biomass yield (g/L).

Eq. (5) was used to calculate the sugar consumption rate (R_S_) (g.L^− 1^.h^− 1^):

5 $$ {\mathrm{R}}_{\mathrm{S}}=\Delta \mathrm{S}/\varDelta \mathrm{t} $$

where ∆S is the amount of sugar consumed (g/L) and ∆t is the fermentation time (h).

### Experimental design and statistical analysis

Factorial designs and response surface methodologies were used to investigate the optimal cultivation parameters affecting fungal biomass growth and oil accumulation. The simultaneous analysis of multiple variables using factorial designs has been shown to increase the accuracy of the results while reducing the overall number of experiments [18]. After the identification of the upstream unit operations needed (enzymatic reactions and heat treatments) to favor fungal growth, a preliminary optimization study using a central composite rotatable design (CCRD) (2^2^, plus 3 central points and 4 axial points) was conducted to identify the individual and combined effects of pH (4.7–6.8) and fermentation time (19.8–90.3 h) on the biomass yield. Fermentations were performed at 34 °C, an intermediate value in the 20–40 °C temperature range commonly accepted to favor the growth of most filamentous fungi [19]. Similar to other findings [20, 21], *M. circinelloides* growth did not take place at temperatures of 36 °C and above.

The results of this preliminary evaluation were used as the basis for another optimization study (CCRD with 2 variables, 3 central points and 4 axial points) where a narrower pH range from 3.60 to 4.7 and temperature values from 22.4 to 33.6 °C were evaluated at 48 and 72 h of fermentation regarding biomass yield and composition. The total number of experiments followed the equation 2^k^ + 2 k + n_c_, where k is the number of independent variables (2) and n_c_ is the number of repetitions in the central point (3). Cultivation temperature and pH, the independent variables, were evaluated according to the following coded levels: - α, − 1, 0, + 1, + α. Coded and uncoded levels and their corresponding independent variables are shown in Table 1. The dependent variables (e.g., evaluated responses) were fungal biomass yield (g of dry biomass/ L), biomass oil content (%, weight of dry biomass) and protein content (%, weight of dry biomass). Data were analyzed by the Protimiza Experiment Design Software (http://experimental-design.protimiza.com.br). The significance of the model was tested by Analysis of Variance (ANOVA).

**Table 1:** Variables and levels evaluated in the experimental design to optimize biomass yield and composition

Experiments	Coded levels		Uncoded levels	
	pH (X_1_)	Temperature (°C) (X_2_)	pH (X_1_)	Temperature (°C) (X_2_)
1	-1	-1	3.8	24.0
2	1	-1	4.5	24.0
3	-1	1	3. 8	32.0
4	1	1	4. 5	32.0
5	−1.41	0	3. 6	28.0
6	+ 1.41	0	4. 7	28.0
7	0	−1.41	4. 1	22.4
8	0	+ 1.41	4. 1	33.6
9	0	0	4. 1	28.0
10	0	0	4. 1	28.0
11	0	0	4. 1	28.0

### Validation of the predictive model

In order to determine the accuracy of the predictive model, a set of shake-flask experiments were performed under the predicted optimal conditions (pH and temperature) at several time points. The validation experiments were conducted in 250 mL Erlyenmyer flasks containing 100 mL aliquots of the HWP. The model validation allowed for further evaluation of the growth kinetics under conditions at which biomass yield and oil content were optimal. All time points were carried out in triplicate and the results are shown as the mean ± one standard deviation.

Lipid quantification and composition was determined for the validation samples at 96, 144 and 168 h. A one-way ANOVA was used to evaluate the effects of fermentation time on biomass yield and composition, lipid class and FA composition during the validation experiment. Generalized linear models from the statistical analysis system (version 9.4, SAS Institute Inc., Cary, NC, USA) were used and comparisons of least-square means were made by Tukey’s adjustment with the level of significance set at *p* < 0.05.

### Fungal biomass and spent media characterization

Monosaccharide composition (glucose and galactose) of the spent media was determined by high-performance anion-exchange chromatography with pulsed amperometric detection (HPAEC-PAD ICS-5000+; Thermo Scientific, Sunnyvale, CA, USA). Calibration curves (R^2^ > 0.999) were prepared with glucose and galactose for simple sugar determination. WP and HWP samples were diluted 10 to 1000 times and filtered through a 0.2-μm membrane. For monosaccharide analysis, a 25-uL aliquot was injected into a Carbo-Pac PA10 (Dionex, Sunnyvale, CA, USA) column at 1.2 mL/min flow rate as described previously [22]. The nitrogen in the spent media and biomass was quantified using the Dumas combustion method (AOAC 990.03) (vario Max cube; Elementar Americas Inc., Ronkonkoma, NY, USA). A nitrogen conversion factor of 6.25 was used to determine the crude protein content [23]. Total oil content of the dried fungal biomass was determined by using the acid hydrolysis Mojonnier method (AOCS method 922.06).

### Lipid profile and fatty acid composition of the fungal biomass oil

To determine total FA concentrations, 20 mg of dry biomass was directly transesterified in methanolic HCl. Samples were mixed with 0.40 mL of toluene spiked with triheptadecanoic acid as an internal standard. Three mL of 100% methanol and 0.60 mL HCl:methanol (8:92 *v*/v) were added in this order and vortexed vigorously. The sample was then incubated at 90 °C for 60 min for derivatization. After cooling to room temperature, 1 mL of hexane and 1 mL of water were added for the extraction of fatty acid methyl esters (FAMEs) and then vortexed. The hexane layer was separated and added to a new centrifuge tube containing 45 mL water. After centrifugation, the top hexane layer containing FAMEs was transferred to a new tube, dried under nitrogen and reconstituted in 0.10 mL hexanes for GC analysis.

To determine the FA composition of the different lipid classes, the lipid fraction of the fungal biomass was first extracted by the Folch extraction method for lipid class analysis. Dry biomass (~ 20 mg) was first sonicated in 3 mL chloroform for 1 min on ice, followed by additional sonication in 2.50 mL chloroform/methanol (2:1, *v*/v) and resuspension in 4.5 mL phosphate-buffered saline. The solvent phase was separated from the biomass by centrifugation (20 min), dried under nitrogen and reconstituted in 0.2 mL chloroform/methanol (2:1, v/v). Lipid classes were separated using thin layer chromatography. Briefly, 0.1 mL of the extract spiked with free fatty acid (FFA) internal standard (17:0) was loaded on a silica plate pre-washed with chloroform/methanol (2:1, v/v). The plate was placed in a tank containing heptane/ethyl ether/ acetic acid (60:40:3, v/v/v). The migration was stopped once the solvent front reached 1–2 cm below the top of the plate. The bands were revealed under UV after spraying the plate with a solution of 0.02% 2′,7′-dichlorofluoroscein in methanol and scraped into new tubes. Fractions containing TAGs, cholesterol esters (CEs) and phospholipids (PLs) were spiked with esterified internal standard (triheptadecanoic acid for TAG and CE; di − 17:0 phosphatidycholine for PL). All fractions were transesterified in methanolic HCl as described above for total FA analysis.

FAMEs were analyzed on a GC Clarus 500 (Perkin Elmer) equipped with a DB-FFAP column (30 m length, 0.25 mm ID, 0.25 um film thickness; Agilent, Santa Clara, CA, USA). The injector and detector temperatures were set at 240 and 300 °C, respectively. For each run, the oven temperature was held at 80 °C for 2 min, increased to 180 °C at 10 °C /min, increased to 240 °C at 5 °C /min and held at 240 °C for 13 min. A custom mix of FAME standards was used to identify the different FAs based on their characteristic retention time.

## Results and Discussion

### Effects of lactose hydrolysis on sugar utilization and biomass yield

The effects of different types of sugars on the biomass yield were investigated by hydrolyzing lactose into glucose and galactose prior to the fermentation step. Sugar utilization was assessed at the level of biomass produced after 72 h of cultivation. Biomass yields of 2.5 and 7.9 g/L were observed for WP and HWP, respectively. The higher biomass yield observed for HWP (~ 3.2 times than that of WP) was coupled with an increased consumption of sugar (9 vs. 86%) when using HWP (see Additional file 1: Figure S1). These results demonstrate that *M. circinelloides* ferments glucose and galactose more efficiently than lactose, with increased biomass yield resulting from monosaccharide utilization. Indeed, Botha et al. [20] reported that *M. circinelloides* could not efficiently utilize disaccharides containing a D-galactopyranosyl or a D-fructofuranosyl moiety. This is likely a consequence of the low expression or activity of enzymes in the *M. circinelloides* strain required to hydrolyze disaccharides such as lactose for effective growth. *M. circinelloides* has been found to contain extracellular β-glucosidases, which not only have the ability to convert cellobiose to glucose, but also hydrolyze the β-glucosidic linkages in lactose. However, the enzyme most likely has a low substrate specificity for lactose since β-glucosidase in *M. circinelloides* was shown to be part of an enzymatic system mainly responsible for cellulose hydrolysis [24]. Our results are in agreement with other studies that observed a low biomass yield when *M. circinelloides* was grown on lactose (1.6 g/L of biomass) as a carbon source compared to that of glucose or galactose (7.0 and 5.3 g/L of biomass) [25].

### Preliminary investigation of the effects of pH and fermentation time on biomass yield

**Fig. 1:**
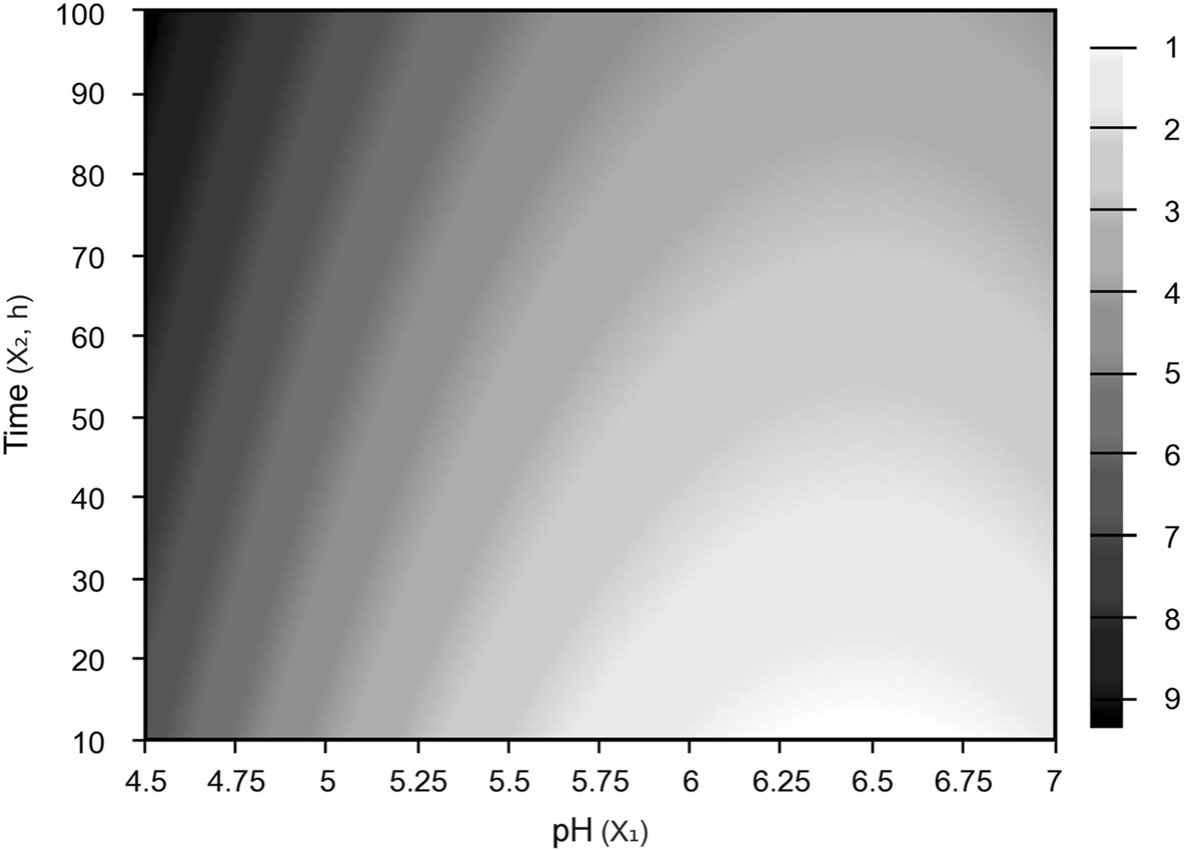
Response surface plot showing the effect of pH and fermentation time on biomass yield

A preliminary optimization was conducted to increase the understanding of the optimal pH range and cultivation time for *M. circinelloides* in HWP. Within the pH range tested (4.7–6.8), low pH values (4.7–5.0) were observed to increase the fungal biomass yield ~ 2.5-fold more than high pH (6.5–6.8) (see Additional file 1: Table S1). Low pH values also enhanced the efficiency of sugar conversion, as demonstrated by the improved biomass yield coefficient (Y_X/S_) values. Additionally, longer fermentation times led to higher biomass formation and increased sugar consumption (> 75%). However, it is important to note that Y_X/S_ decreased with time, most likely due to the cell growth rate slowing down over the time course of the fermentation. The effects of pH and time levels evaluated were determined by multiple regression analysis of the experimental data. Only parameters significant at *p* < 0.05 were included in the estimated regression model. The regression equation of second order shows the dependence of biomass yield (Y_1_) to pH and time (Eq. 5), while the sugar consumption (Y_2_) has been shown to depend only on fermentation time (Eq. 6):

5 $$ {\mathrm{Y}}_1=3.03-1.58{\mathrm{X}}_1+0.82{{\mathrm{X}}_1}^2+0.81{\mathrm{X}}_2 $$

6 $$ {\mathrm{Y}}_2=51.71+24.10{\mathrm{X}}_2 $$

where X_1_ and X_2_ are the independent variables of pH and time, respectively. The coefficients of determination (R^2^) for biomass yield and sugar consumption were able to explain 93 and 87% of the variation between the predicted and experimental data, respectively. The regression was significant (F_calculated_ > F_tabulated_) with no lack of fit being observed for the regression models. Based on the regression model, a surface contour was built to describe the combined effects of pH and fermentation time on the biomass yield (Fig. 1). According to Fig. 1, biomass yield is favored by lower pH values and longer fermentation times, with maximum biomass yield achieved at pH 4.5 and a fermentation time of 90 h. These results demonstrated that a low pH was optimal for biomass production from pasteurized HWP, likely due to reduced bacterial competition during fermentation [26].

### Synergistic effects of pH and thermal treatments on fungal biomass yield

Autoclaving is a typical practice used to sterilize media before fungal fermentation [6, 16, 27]. Although autoclaving effectively eliminates most microbes, it is an energy intensive and costly process considering the high production volume of agricultural streams that can be used as cultivation mediums. Therefore, using alternative sterilization methods such as high temperature short time pasteurization (HTST) may be more economic and energy efficient.

To evaluate the potential of replacing autoclaving with HTST pasteurization in such protocols, we compared the fermentation results obtained with pasteurization (72 °C, 15 s) to those obtained with sterilization (121 °C, 20 min) using pH (4.5 and 6.5). According to the preliminary assessment of the effect of pH on biomass yield, fermentation of pasteurized HWP at pH 4.5 resulted in increased fungal biomass yield (6.5 g/L) compared to that of pH 6.5 (3.0 g/L). Using autoclaved HWP, fermentations conducted at pH 4.5 and 6.5 both resulted in a fungal biomass yield of 6.3 g/L. Consequently, the biomass yield obtained from low pH and pasteurization (6.5 g/L) was comparable to those obtained under aseptic conditions (6.3 g/L). These results indicated that the use of low pH (4.5) combined with standard HTST pasteurization (72 °C, 15 s) might reduce bacterial growth in the medium, as evidenced by the increased capacity of *M. circinelloides* to thrive and produce an increased biomass yield.

To corroborate those findings, the viable cell counts of the pasteurized spent media (post-fermentation) at pH 4.5 and 6.5 were determined. Total bacteria counts were higher at pH 6.5 (5.8 × 10^4^ CFU mL^− 1^) than that of pH 4.5 (1 × 10^1^ CFU mL^− 1^), demonstrating that the use of low pH inhibits bacterial growth, thus favoring fungal biomass accumulation. Consequently, the use of low pH may enable the replacement of sterilization of the medium with pasteurization, a well-established unit operation in the dairy industry. Limited studies have investigated the fungal conversion of food by-products under non-aseptic conditions. Specifically, Moustogianni et al. [28] achieved suppressed bacterial contamination of non-aseptic oleaginous Zygomycetes cultures using a combination of low pH (4.0) and antibacterial agents such as essential oils. Tchakouteu et al. [29] reached similar results in oleaginous yeast cultures using pasteurized media with the addition of NaCl. However, our study provides a novel method of non-aseptic fermentation that omits the cost of additional media supplementation (e.g. antibacterial agents). In this case, the use of pH 4.5, in conjunction with pasteurization, not only reduced bacterial counts in the culture, but also resulted in a cell growth of *M. ciricnelloides* that was comparable to that of aseptic conditions. These findings could lead to substantial energy savings in terms of processing cost reduction based on the elimination of sterilization prior to fermentation.

### Optimization of fungal biomass yield and composition

Temperature and pH are two important reaction parameters affecting fungal growth and biomass composition. Because fungal biomass growth was shown to be favored by a lower pH, the simultaneous interaction of temperature and a narrower pH range (3.6 to 4.7) was evaluated using a central composite rotatable design. In order to identify possible improvements in the fermentation rate during the experimental design, shorter fermentation times (48 and 72 h) were evaluated for each experimental condition, with total biomass and supernatant collected at both time points.

**Fig. 2:**
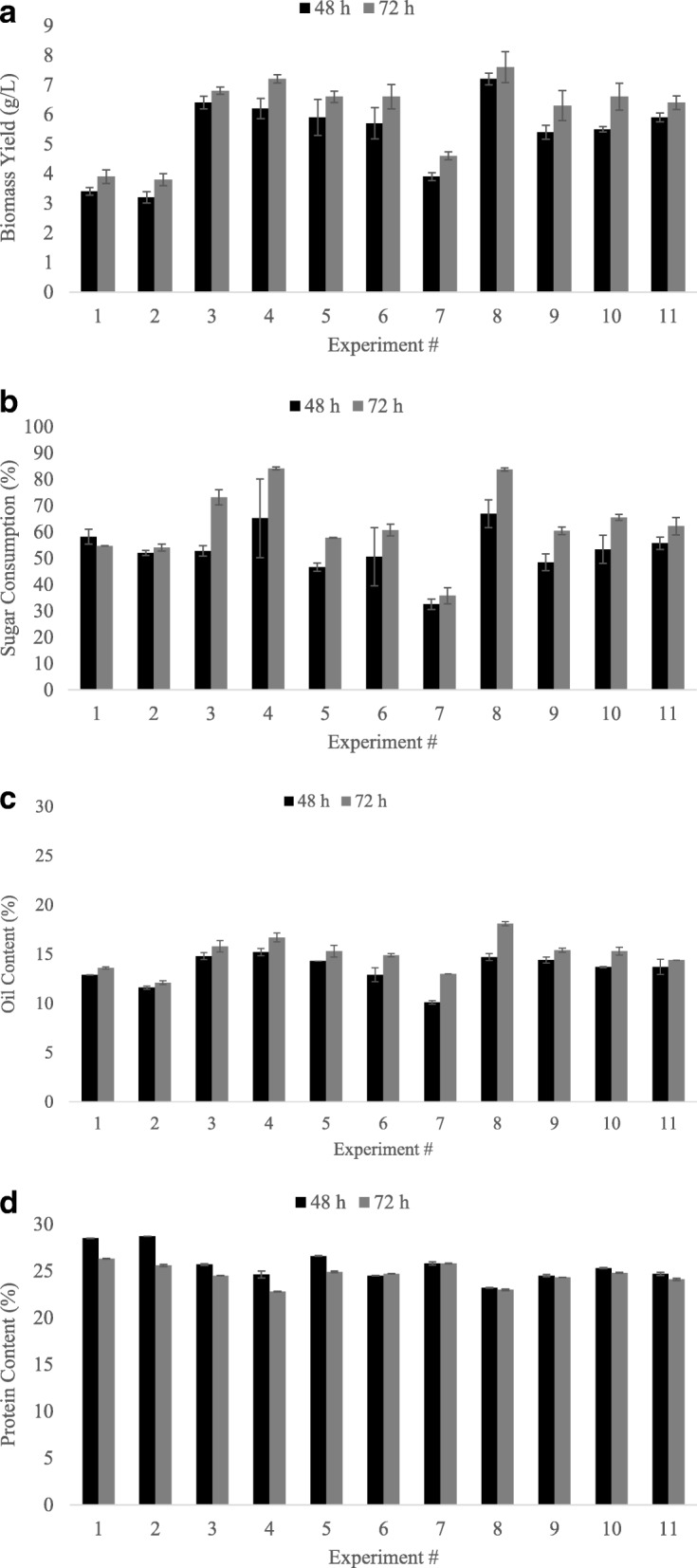
Experimental results obtained in the optimization. Results are shown for the biomass yield (g/L) (**a**), sugar consumption (%) (**b**), oil content (%, *w*/w_DB_) (**c**) and protein content (%, *w*/w_DB_) (**d**) at 48 and 72 h of fermentation. w/w_DB_ is the mass of the component (oil or protein)/mass of dry biomass. Temperature and pH of each experimental run: 1 = 24 °C, 3.8; 2 = 24 °C, 4.5; 3 = 32 °C, 3.8; 4 = 32 °C, 4.5; 5 = 32 °C, 4.5; 6 = 28 °C, 4.5; 7 = 28 °C, 4.7; 8 = 22.4 °C, 4.1; 9 = 33.6 °C, 4.1; 10 = 33.6 °C, 4.1; 11 = 33.6 °C, 4.1

The effects of temperature and pH on biomass yield, sugar consumption, oil accumulation and protein content are shown in Fig. 2a, b, c and d, respectively. As observed in Fig. 2a, higher biomass yields (6.8–7.6 g/L) were observed at experimental conditions where temperatures above 28 °C were used (experiment #3, 4, and 8). However, the acidic pH range (3.6–4.7) used had a less pronounced effect on biomass yield, indicating that the pH values evaluated were already within the optimum range explored in the preliminary optimization. Biomass yield increments ranging from 6.89 to 17.17% were observed by increasing the fermentation time from 48 to 72 h. Experiments #3, 4, and 8 were subsequently correlated with higher sugar consumption in the spent media (73–84%) (Fig. 2b). This demonstrates that this fungal strain consumes sugars at a faster rate at temperatures above 28 °C, evidencing the effect of temperature on fungal metabolism and growth. However, full consumption of sugars was not achieved at the longest fermentation time (72 h), indicating that fungal growth was not yet completed. Likewise, oil content in the biomass was also favored by longer fermentation times and higher temperatures. By increasing the fermentation time from 48 to 72 h, the oil content had a percent increase ranging from 4.13 to 22.08%. Highest intracellular oil contents (15.8–18%) were observed for experiments 3, 4, and 8, where higher temperature values were used (Fig. 2c).

The maximum biomass yield (7.6 g/L) and oil content (18%) was observed at 72 h at 33.6 °C. Our results are in agreement with the literature, where small increments in cultivation temperatures within a certain range resulted in increased biomass yield and lipid content. Xia et al. [30] reported an optimal growth range between 24 and 30 °C for *M. circinelloides* grown in glucose-containing media, with the total lipid content significantly increasing from about 10 to over 22% when the temperature reached 35 °C. Likewise, the cultivation of *Mortierella ramanniana* at 30 °C led to higher lipid accumulation compared to that of lower cultivation temperatures [31]. These results indicate that high temperature facilitates lipid accumulation, independent of nitrogen depletion. Similarly, the *M. circinelloides* oil content reported herein increased when exposed to high temperature, implying that temperature may serve to induce lipid synthesis. In some cases of extreme temperatures, stress conditions can inhibit cell growth and redirect the available nutrient for lipid accumulation, therefore leading to a decreased biomass yield [30]. However, our results show that an increase in oil content was also paralleled with an increase in biomass yield. This suggests that the temperature range evaluated in our study (22.4–33.6 °C) did not include extreme temperatures that could lead to the suppression of cell growth.

According to Fig. 2d., the *M. circinelloides* biomass contained a higher protein content than that of oil within the fermentation time evaluated (48–72 h), which is in agreement with several studies using filamentous fungi. Satari et al. [32] found that *Mucor indicus* produced a biomass containing 40% protein and only 10% oil under optimal conditions in corn waste free sugars. Similarly, the cultivation of *Rhizopus oligosporus* and *Neurospora intermedia* in thin stillage resulted in a biomass containing 43 and 50% crude protein and 20 and 12% oil, respectively [4, 33]. However, our results also demonstrated a small reduction in the biomass protein content at higher fermentation temperatures (≥ 32 °C). An average protein content of 23% was observed in experiments #3, 4, and 8 at 72 h compared to that of the runs conducted at ≤24 °C (~ 26%). These results suggest that while higher temperatures seem to improve lipid accumulation, it is at the expense of protein.

### Statistical analysis for the optimization of biomass yield and composition

Since pH values were already within the optimized range revealed from the preliminary tests, temperature was shown to be the only parameter having a statistically significant effect on biomass yield and intracellular oil. Because higher biomass yields and oil contents were obtained at 72 h, only the estimated regression models for biomass yield (g/L), oil and protein contents (%) at 72 h are shown in Table 2. For all cases, the regression was significant (F_calculated_ > F_tabulated_), while the F-test for lack of fit was not statistically significant. This indicates that the models do not show lack of fit and can be used for predictive goals in the range of the parameters evaluated [18].

**Table 2:** Analysis of variance (ANOVA) of the estimated regression models for biomass yield, sugar consumption, and oil and protein contents in the fungal biomass at 72 h

Estimated regression models	R^2^	F_cal_	F_tab_
Estimated biomass yield (g/L) = 6.04 + 1.32 X_2_	0.82	40.6	5.12
Estimated sugar consumption (%) = 62.92 + 14.56X_2_	0.88	68.8	5.12
Estimated oil content (%) = 14.97 + 1.75X_2_	0.87	61.1	5.12
Estimated protein content (%) = 24.62–1.07X_2_	0.78	31.5	5.12

Only parameters significant at *p* < 0.05 were used in the regression models. X_2_ is the coded level corresponding to temperature variable, F_cal_ is the calculated F-ratio (MS regression/MS residual) and F_tab_ is the tabulated F-value (0.95, df regression, df residual), where MS is mean square and df is degrees of freedom

The optimal level for each independent variable evaluated was determined by multiple regression analysis of the experimental data. Only parameters significant at *p* < 0.05 were used in the regression models. The regression equation of second order shows the dependence of the biomass yield to temperature at 72 h, where X_2_ is the independent variable temperature. The R^2^ of the predictive model for biomass yield at 72 h was 0.82. This indicates that the regression model was able to explain 82% of the total variation between the observed and predicted values with the remaining 18% being attributed to the residual values. According to the estimated regression model, biomass yield increases when the temperature value increases from − 1.41 to + 1.41 (22.4–33.6 °C), for any of the pH levels tested. Similarly, oil and protein contents showed a dependence on temperature. The R^2^ of the predictive models for biomass oil and protein content were 87.1 and 77.8%, respectively, indicating that 12.8 and 22.2% of the total variations were not explained by the model, thus being attributed to the residual values.

The independent variable pH was not statistically significant in the range evaluated (3.6–4.7) and therefore was not included in the models. According to the estimated regression models, optimum condition for increased biomass yield, higher oil content and sugar consumption can be achieved at temperature of 33.6 °C (+ 1.41). The regression models demonstrate a positive linear relationship with temperature. Under the optimum temperature, within the conditions tested, the values for biomass yield, lipid accumulation and sugar consumption were 7.63 g/L, 18.09 and 83.37%, respectively, which were close to the predicted responses of 7.9 g/L, 17.44% and 83.45%, indicating the applicability of the proposed model. A decrease in sugar concentration in HWP during fermentation suggests effective substrate uptake for better fungal growth and/or oil accumulation, as seen in previous reports [25, 34]. However, the estimated regression model for protein accumulation in the fungal biomass demonstrates a negative correlation between higher temperature and protein accumulation, with reduced protein content observed at higher temperatures.

### Validation of the predictive model at laboratory scale

**Fig. 3:**
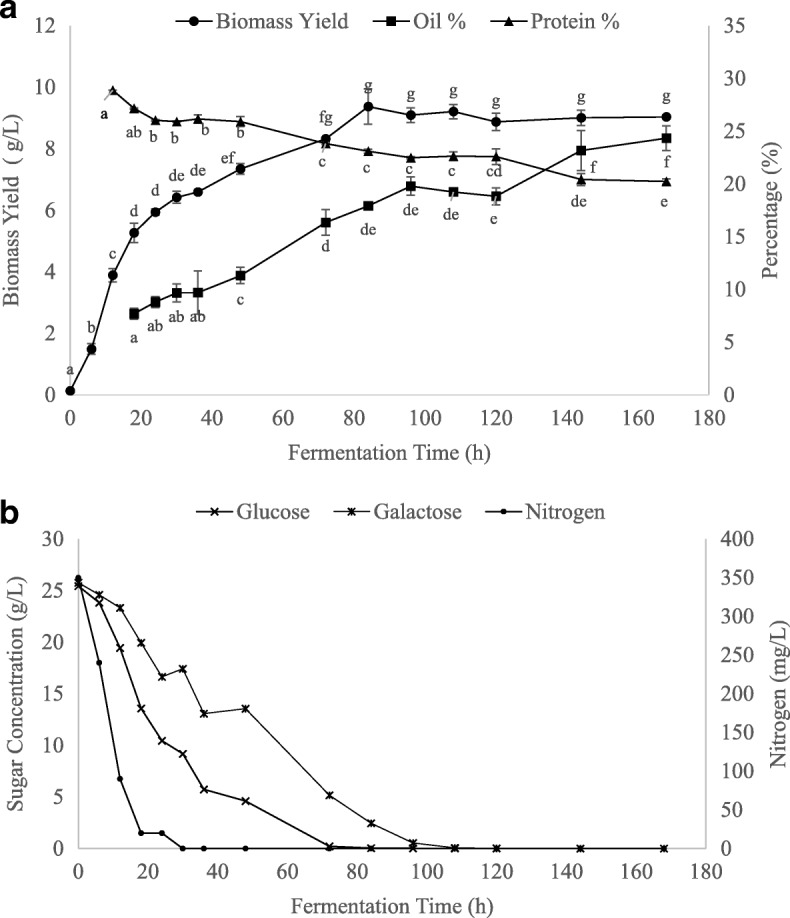
**a** Biomass yield and composition and **b** sugar and nitrogen concentration in spent media produced from the validation experiment. Fermentations conducted at optimal conditions (pH 4.5, 33.6 °C) for 7 days. Oil and protein content expressed as %, w/w_DB_, where w/w_DB_ is the mass of the component (oil or protein)/mass of dry biomass. Values sharing the same letters are not statistically different at *p* < 0.05

To validate the predictive model for biomass yield and oil content, the best processing conditions identified in the experimental design were conducted in triplicate. Fermentations were carried out at 33.6 °C and pH 4.5. Since pH within the values of 3.6–4.7 was shown to not be a significant variable, the pH value of 4.5 was selected because it favors efficient lactose hydrolysis prior to fermentation. Sugar and nitrogen concentration of the spent media were measured to better understand substrate utilization and its impact on biomass yield and composition, as seen in Fig. 3a and b. The growth kinetics were evaluated by withdrawing samples for 7 days. Table 3 shows several parameters including biomass (X) and lipid (P) yields, together with yield coefficients (Y_X/S_, Y_P/S_, and Y_P/X_).

**Table 3:** Biomass and lipid yields and yield coefficients obtained for *M. circinelloides* grown at optimal conditions

t (h)	X (g/L)	P (g/L)	Y_X/S_ (g/g)	Y_P/S_ (g/g)	Y_P/X_ (g/g)	R_S_ (g.L^−1^.h^−1^)
24	5.93 ± 0.54	0.52 ± 0.03	0.25 ± 0.06	0.02 ± 0.01	0.09 ± 0.01	1.00 ± 0.26
48	7.34 ± 0.15	0.83 ± 0.00	0.22 ± 0.01	0.03 ± 0.00	0.11 ± 0.00	0.69 ± 0.02
72	8.32 ± 0.30	1.36 ± 0.07	0.18 ± 0.01	0.03 ± 0.00	0.16 ± 0.00	0.64 ± 0.00
96	9.09 ± 0.91	1.80 ± 0.37	0.18 ± 0.02	0.04 ± 0.01	0.20 ± 0.01	0.53 ± 0.00
120	8.87 ± 0.47	1.67 ± 0.19	0.17 ± 0.01	0.03 ± 0.00	0.19 ± 0.00	0.43 ± 0.00
144	9.00 ± 0.50	2.09 ± 0.21	0.18 ± 0.01	0.04 ± 0.00	0.23 ± 0.00	0.36 ± 0.00
168	9.03 ± 0.74	2.20 ± 0.25	0.18 ± 0.01	0.04 ± 0.00	0.24 ± 0.00	0.30 ± 0.00

Representation of biomass (*∆*X) and lipid yields (*∆*P), coefficient of biomass yield to sugar consumption (Y_X/S_, g/g), coefficient of lipid yield to sugar consumption (Y_P/S,_ g/g), coefficient of lipid yield to biomass yield (Y_P/X,_ g/g), and rate of sugar consumption (R_S_, g.L^−1^.h^−1^). Fermentations conducted at pH 4.5 and 33.6 °C, with initial sugar concentration in hydrolyzed whey permeate at 51.2 g/L. Data is the mean ± standard deviation of three replicates

Maximum biomass production of 9.37 g/L was observed at approximately 80 h, after which the biomass yield did not significantly change (Fig. 3a). Simple sugars (glucose, galactose) in the culture media were incorporated by 72–96 h (Fig. 3b). Glucose was consumed preferentially by *M. circinelloides*, with full fermentation of glucose accomplished within 72 h. However, the simultaneous assimilation of both sugars occurred, suggesting the absence of diauxic growth of the fungus. This occurrence is further illustrated by the biomass growth curve that continued without visible disturbance. Similarly, Lübbehüsen et al. [35] reported that *M. circinelloides* was able to switch rapidly from one sugar to another when cultivated in a mixture of glucose and xylose. Consequently, *M. circinelloides* may have the capacity to readily adapt to a new carbon source. Moreover, the nitrogen in HWP was quickly exhausted within 24 h, corresponding to the cells leaving exponential phase (see Additional file 1: Figure S2). The observed increase in biomass yield after exponential phase in Fig. 3a may be due to the cells accumulating oil as a secondary metabolite, rather than an increase in cell number. This is corroborated by the calculated lipid-free biomass (∆X − ∆P), which increases to a much lesser extent during this stage of fermentation (data not shown). Likewise, Meeuwse et al. [36] found that the lipid-free biomass yield of *Motierella isabellina* remained relatively constant after exponential phase. Nitrogen depletion, combined with excess sugar supply, most likely shifted fungal metabolic activities from rapid cell growth toward de novo lipid accumulation. The total biomass yield in our study plateaued after sugar exhaustion at 72–96 h, while the lipid content of the fungal cells increased to 24%. This trend is supported by the specific lipid yield coefficient (Y_P/X_), which shows that the lipid amount in the biomass continued to increase throughout the fermentation, despite the biomass yield becoming stationary. The underlying mechanisms of nitrogen depletion in a fermentation medium leading to FA accumulation has been suggested for *M. circinelloides.* Song et al. [37] discovered six isoforms of malic enzyme (ME) in *M. circinelloides*, with only isoform IV appearing under nitrogen starvation conditions to provide NADPH for lipid accumulation. A study conducted by Zhang et al. [38] further found that overexpression of the gene encoding ME isoforms III/IV in *M. circinelloides* led to a 2.5-fold increase in total lipid content. Consequently, nitrogen depletion in HWP may have triggered enzymatic changes to induce lipid biosynthesis under non-growth conditions, leading to an observed increase in oil content [39, 40].

According to Table 3, the lipid yield increased from 0.52 to 2.20 g/L with fermentation time, while the biomass yield coefficient, Y_X/S_, remained constant at approximately 0.18 g/g after 72 h, which coincides with almost complete sugar depletion. At around 168 h of fermentation, the highest lipid yield (2.20 ± 0.25 g/L) was observed, which corresponds to a lipid content of 24% in dry biomass. This suggests that prolonged nutrient-starvation conditions resulted in a statistically significant higher lipid accumulation in the strain used in our study. Interestingly, reserve lipid turnover was not observed after transition from carbon excess to carbon starvation conditions. Papanikolaou et al. [41] found that reserve lipid in *Cunninghamella echinulata* was not degraded after glucose exhaustion, suggesting that reserve lipid turnover in oleaginous fungi could be repressed in multiple-limited media. Thus, the absence of lipid turnover might be related to the lack of sufficient concentrations of several nutrients in HWP (e.g. vitamins, trace minerals or metalloids) that are essential for functioning the biochemical mechanisms involved in the mobilization of reserve lipid. For example, lipases need metallic co-factors for activation while magnesium is crucial for the action of isocitrate lyase, an enzyme involved in FA degradation [41, 42]. Additionally, several strains of oleaginous yeast such as *Cryptococcus curvatus* and *Yarrowia lipolytica* have been found to accumulate intracellular polysaccharides in nitrogen-excess conditions, which may be degraded in the late fermentation stages in favor of storage lipids [43, 44]. The utilization of storage polysaccharides under nutrient starvation conditions may further explain the increasing accumulation of lipids in the *M. circinelloides* biomass in our study despite sugar depletion occurring in HWP.

The lipid yield achieved in this study (1.36 g/L) at 72 h was similar to findings observed from the fermentation of *M. circinelloides* strain NRRL3631, which had a lipid yield of 1.60 g/L when grown in ricotta cheese whey medium for 72 h [17]. Vicente et al. [45] found that the genetically modified *M. circinelloides* strain MU241 had a lipid content of 22.9% in dry biomass, with a lower lipid yield of 0.96 g/L when cultivated for 96 h. Although *M. circinelloides* has been extensively studied for its oil accumulation, its lipid production in our study is reduced compared with other recently studied fungal strains [6, 46, 47]. This may be due to the lack of supplementation of the HWP, causing the fermentation to start with a lower quantity of nutrients. Although enhancement of lipid production may be observed by adding an external carbon source or microelements, the addition of nutrients for microbial fermentations is associated with higher processing costs [48, 49].

It is also important to note that a decrease in the protein content is paralleled by an increase in oil content during fungal growth and stationary phase. The oil content in dry biomass reached a maximum of 24%, while protein reached a minimum of 20.22%. For oleaginous microorganisms, it has been reported that the lack of nitrogen limits the capacity to synthesize proteins and nucleic acids necessary for biomass production [50]. In order to compensate, *M. circinelloides* may have taken advantage of alternative metabolic pathways for inorganic carbon fixation such as FA synthesis, and hence, store those de novo FAs as TAG. Consequently, nitrogen starvation within 30 h may have imposed a reduction in cellular protein content and inhibited the growth rate.

### Lipid class and fatty acid profile

The biomass lipid was extracted from dry biomass harvested at 96, 144 and 168 h. Lipid classes in the corresponding samples were separated by thin layer chromatography and FA profiles were determined by gas chromatography. The lipid distribution of TAG, PL, CE and FFA, expressed as a percentage of the summed total, are presented in Table 4. It should be noted that the summed total of FAs derived from TAGs, CEs, PLs and FFAs extracted by the Folch method was less than the total measured by direct acid transesterification (see Additional file 1: Table S2). This is expected since acid hydrolysis can degrade the fungal cell wall structures and transesterify TAGs and other lipids. However, the Folch solvent does not degrade the cell wall, which is why lipid accessibility and extraction is limited.

**Table 4:** Lipid distribution of the intracellular oil after 96, 144 and 168 h of fermentation at optimal conditions

Fermentation Time (h)			
Lipid Distribution (%)	96	144	168
TAG	88.5^a^	90.9^a^	92.1^a^
CE	0.7^a^	1.5^a^	0.4^a^
PL	5.8^a^	4.9^a^	4.5^a^
FFA	4.9^a^	2.7^b^	3.0^b^

Values that share the same superscript are not significantly different at *p* < 0.05

*TAG* Triacylglycerides, *CE* Cholesterol esters, *PL* Phospholipids and FFA-free fatty acids

According to Table 4, the majority of FAs were esterified into TAG molecules, which is similar to the lipid profile of most oleaginous fungi. Fungi store a large proportion of their energy carbon as neutral lipids, thus the amount of neutral lipids is usually higher than that of PLs for membrane constituents [51]. Likewise, Fakas et al. [52] reported TAG to be the major constituent of the lipid extracted from *C. echinulata* cultivated on tomato waste hydrolysate, accounting for 90% of the total lipid, while FFAs and sterols were present in lower quantities. It was observed in our study that the percentage of lipid distributed into TAGs increased during fermentation time. It was also accompanied by a significant decrease in FFA at 144 and 168 h compared to that of 96 h, possibly due to the assimilation of FFA into the TAG-synthesis pathway over time. However, there seemed to be no significant differences in fermentation times for the amount of CE and PLs in the oil.

As seen in Table 5, oleic acid (C18:1-cis) and palmitic acid (C16:0) were the predominant FA components in the *M. circinelloides* oil. Linoleic (C18:2n-6), γ-linolenic acid (C18:3(n-6)) and palmitoleic acid (C16:1) were found in smaller quantities while myristic (C14:0) and stearic acid (C18:0) were detected in the lowest amounts. The fermentation time of *M. circinelloides* was not accompanied by any changes in FA composition of TAG. Harsh environmental conditions, such as high temperature and pH, might have a greater influence on FA composition compared to fermentation time [13, 53].

**Table 5:** Major fatty acids of triglycerides in *M. circinelloides* oil after 96, 144 and 168 h of fermentation at optimal conditions

Fermentation Time (h)			
Fatty Acid (%, w/w^1^)	96	144	168
C14:0	3.0^a^	3.0^a^	3.0^a^
C16:0	21.0^a^	21.0^a^	23.0^a^
C16:1	10.0^a^	8.0^a^	7.0^a^
C18:0	3.0^a^	3.0^a^	4.0^a^
C18:1 (cis)	43.0^a^	42.0^a^	41.0^a^
C18:2 (n-6)	7.0^a^	10.0^ab^	11.0^b^
C18:3 (n-6)	9.0^a^	10.0^a^	9.0^a^

Values that share the same superscript are not significantly different at *p* < 0.05

^1^Relative percentage (weight/weight) of the total fatty acid groups

The FA composition of *M. circinelloides* lipid grown in HWP was comparable to that of other oils produced from zygomycetes fungi [49, 54, 55]. Carvalho et al. [5] conducted a comparison of microbial oil from *M. circinelloides* and palm oil commonly used for biofuel, showing that both oils have similar oleic acid content (39%) and monounsaturated FA composition. In particular, palmitic acid (C16:0), and oleic acid (C18:1), which are the predominant FAs observed in our oil, are potential targets of interest due to their oxidative stability and potential adaptability in the industrial production of biodiesel [56]. The total lipids from *M. circinelloides* oil measured in our study were saponifiable lipids and FFAs, which can be easily converted to FAMEs for high quality biodiesel [57]. Additionally, the oil in our study contained an adequate amount of γ-linolenic acid (10%), which is an omega-6 FA found mostly in plant-based oils and can be used to supplement dietary intakes [13]. The total amount of GLA synthesized at 96, 144 and 168 h of fermentation was approximately 53.4, 73.4 and 69.4 mg/L, respectively. The GLA concentration in our strain is lower than that of other *M. circinelloides* strains investigated in the literature, which were cultivated in vegetable oils and vitamin enriched mediums as opposed to simple sugars [58–60]. Additionally, several strains of Mucor spp., such as *Mucor rouxii* were found to increase GLA concentration during cellular oil degradation. According to Aggelis et al. [61] and Kavadia et al. [54], the reserve lipid may be degraded to produce fat-free biomass and provide the growing mycelial membrane with the necessary amount of GLA. If lipid degradation was achieved in our study within the fermentation time tested, a higher GLA concentration may have been produced. Overall, the lipid composition suggests that the lipids are potentially suitable for second generation biodiesel production.

## Conclusions

*Mucor circinelloides* produced oil when grown on cheese HWP. Response surface methodology was useful to determine the optimum pH and temperature, within the range evaluated, for increased biomass yield and oil accumulation. Similar to other oleaginous species, biomass yield and lipid accumulation of *M. circinelloides* was triggered by high temperature, while a low pH (4.5) helped decrease microbial competition during fermentation. Maximum biomass yield of 9.4 g/L and lipid content of 24% were achieved at optimal condition of 33.6 °C and pH 4.5 during 168 h of fermentation. Oleic and palmitic FAs were predominant in the lipid fraction, suggesting the possible use of *M. circinelloides* oil as food or as a feedstock for biodiesel production. In addition, the relatively high content of γ-linolenic acid might enable the use of this oil for nutraceutical applications. This study demonstrated that the use of *Mucor circinelloides* is a viable approach to convert the high organic load in HWP into value-added compounds such as oil. This signifies a starting point for further studies aimed at assessing the development of a fully functioning fungi-to-food/fuel system on an industrial scale for several agricultural streams.

## Additional file

Additional file 1: **Figure S1.** Effect of lactose hydrolysis on biomass yield and sugar consumption. *M. circinelloides* was grown on whey permeate and hydrolyzed whey permeate at pH 4.5 and 34 °C for 72 h. **Figure S2**. Plot of the natural log of biomass yield (g/L) versus fermentation time. Fermentations conducted at pH 4.5 and 33.6 °C at shake-flask. **Table S1.** Preliminary experimental design for optimizing pH and fermentation time to improve fungal biomass yield. **Table S2**. Total fatty acid concentration (nmol/g) measured by the Folch extraction method versus the acid hydrolysis method. (DOCX 30 kb)

## Abbreviations

∆P: Lipid yield
∆X: Biomass yield
ANOVA: Analysis of variance
BOD: Biological oxygen demand
CE: Cholesterol ester
C_L_: Cellular lipid content
COD: Chemical oxygen demand
FA: Fatty acid
FAME: Fatty acid methyl ester
FFA: Free fatty acid
HTST: High temperature short time pasteurization
HWP: Hydrolyzed whey permeate
ME: Malic enzyme
PL: Phospholipid
R^2^: Coefficient of determination
R_s_: Rate of sugar consumption
TAG: Triacylglyceride
Y_P/S_: Lipid yield coefficient
Y_P/X_: Specific lipid yield coefficient
Y_X/S_: Biomass yield coefficient
