# Supplementary material for: Bioconversion of cheese whey permeate into fungal oil by Mucor circinelloides
Source: J Biol Eng. 2018 Nov 14;12:25. doi: 10.1186/s13036-018-0116-5 (PMC6237013; doi:10.1186/s13036-018-0116-5)
Supplement: Supplementary file 1 — Figure S1. Effect of lactose hydrolysis on biomass yield and sugar consumption. M. circinelloides was grown on whey permeate and hydrolyzed whey permeate at pH 4.5 and 34 °C for 72 h. Figure S2. Plot of the natural log of biomass yield (g/L) versus fermentation time. Fermentations conducted at pH 4.5 and 33.6 °C at shake-flask. Table S1. Preliminary experimental design for optimizing pH and fermentation time to improve fungal biomass yield. Table S2. Total fatty acid concentration (nmol/g) measured by the Folch extraction method versus the acid hydrolysis method. (DOCX 30 kb) [file 13036_2018_116_MOESM1_ESM.docx]

**Additional file 1**

**for**

**Bioconversion of Cheese Whey Permeate into Fungal Oil by *Mucor circinelloides***

Lauryn G. Chan^1^, Joshua L. Cohen^1^, Gulustan Ozturk^1^, Marie Hennebelle^1^, Ameer Y. Taha^1^, Juliana M. L. N. de Moura Bell ^1,2*^

^1^Department of Food Science and Technology, University of California, Davis, One Shields Avenue, Davis, CA, 95616, United States

^2^ Department of Biological and Agricultural Engineering, Davis, One Shields Avenue, Davis, CA, 95616, United States

*corresponding author: jdemourabell@ucdavis.edu

Supplementary Figures

**Figure S1.** Effect of lactose hydrolysis on biomass yield and sugar consumption. *M. circinelloides* was grown on whey permeate and hydrolyzed whey permeate at pH 4.5 and 34 ^o^C for 72 h.

Supplementary Figures

**Figure S2**. Plot of the natural log of biomass yield (g/L) versus fermentation time. Fermentations conducted at 33.6 °C and pH 4.5 at shake-flask.

Supplementary Tables

**Table S1.** Preliminary experimental design for optimizing pH and fermentation time to improve fungal biomass yield

| **Treatments** | **pH**  **(X_1_)** | **Time (h)**  **(X_2_)** | **Biomass Yield**  **(g/L)** | **Sugar consumption**  **(%)** | **Y_X/S_** |
| --- | --- | --- | --- | --- | --- |
| 1 | 5.0 | 30.0 | 4.4 | 22.6 | 0.38 |
| 2 | 6.5 | 30.0 | 1.6 | 32.8 | 0.09 |
| 3 | 5.0 | 80.0 | 6.9 | 76.3 | 0.17 |
| 4 | 6.5 | 80.0 | 2.8 | 77.7 | 0.07 |
| 5 | 4.7 | 55.0 | 6.5 | 59.5 | 0.21 |
| 6 | 6.8 | 55.0 | 3.0 | 33.3 | 0.17 |
| 7 | 5.8 | 19.8 | 1.7 | 14.6 | 0.22 |
| 8 | 5.8 | 90.3 | 4.0 | 81.1 | 0.09 |
| 9 | 5.8 | 55.0 | 3.1 | 53.8 | 0.11 |
| 10 | 5.8 | 55.0 | 3.5 | 64.9 | 0.11 |
| 11 | 5.8 | 55.0 | 3.1 | 52.4 | 0.12 |

Supplementary Tables

**Table S2**. Total fatty acid concentration (nmol/g) measured by the Folch extraction method versus the acid hydrolysis method.

| Time (h) | Folch Extraction | Acid Hydrolysis |
| --- | --- | --- |
| 96 | 244251.1 | 445428.9 |
| 144 | 327665.6 | 476780.0 |
| 168 | 307810.9 | 430266.8 |
